# In chronic myeloid leukemia patients on second-line tyrosine kinase inhibitor therapy, deep sequencing of BCR-ABL1 at the time of warning may allow sensitive detection of emerging drug-resistant mutants

**DOI:** 10.1186/s12885-016-2635-0

**Published:** 2016-08-02

**Authors:** Simona Soverini, Caterina De Benedittis, Fausto Castagnetti, Gabriele Gugliotta, Manuela Mancini, Luana Bavaro, Katerina Machova Polakova, Jana Linhartova, Alessandra Iurlo, Domenico Russo, Fabrizio Pane, Giuseppe Saglio, Gianantonio Rosti, Michele Cavo, Michele Baccarani, Giovanni Martinelli

**Affiliations:** 1 Hematology “L. e A. Seràgnoli”, Department of Experimental, Diagnostic and Specialty Medicine, University of Bologna, Bologna, Italy; 2 Institute of Hematology and Blood Transfusion, Prague, Czech Republic; 3 Division of Haematology, Fondazione IRCCS Ca’ Granda Ospedale Maggiore Policlinico, Milan, Italy; 4 Unit of Blood Disease and Stem Cell Transplantation, Department of Clinical and Experimental Sciences, University of Brescia, Brescia, Italy; 5 Department of Biochemistry and Medical Biotechnologies, University of Naples Federico II, Naples, Italy; 6 Department of Clinical and Biological Sciences “S. Luigi Gonzaga” Hospital, University of Turin, Orbassano, Italy; 7 Institute of Hematology “L. e A. Seràgnoli”, Via Massarenti 9, 40138 Bologna, Italy

**Keywords:** BCR-ABL1, Chronic myeloid leukemia, Tyrosine kinase inhibitors, Warning, Deep sequencing

## Abstract

**Background:**

Imatinib-resistant chronic myeloid leukemia (CML) patients receiving second-line tyrosine kinase inhibitor (TKI) therapy with dasatinib or nilotinib have a higher risk of disease relapse and progression and not infrequently BCR-ABL1 kinase domain (KD) mutations are implicated in therapeutic failure. In this setting, earlier detection of emerging BCR-ABL1 KD mutations would offer greater chances of efficacy for subsequent salvage therapy and limit the biological consequences of full BCR-ABL1 kinase reactivation. Taking advantage of an already set up and validated next-generation deep amplicon sequencing (DS) assay, we aimed to assess whether DS may allow a larger window of detection of emerging BCR-ABL1 KD mutants predicting for an impending relapse.

**Methods:**

a total of 125 longitudinal samples from 51 CML patients who had acquired dasatinib- or nilotinib-resistant mutations during second-line therapy were analyzed by DS from the time of failure and mutation detection by conventional sequencing backwards. BCR-ABL1/ABL1%^IS^ transcript levels were used to define whether the patient had ‘optimal response’, ‘warning’ or ‘failure’ at the time of first mutation detection by DS.

**Results:**

DS was able to backtrack dasatinib- or nilotinib-resistant mutations to the previous sample(s) in 23/51 (45 %) pts. Median mutation burden at the time of first detection by DS was 5.5 % (range, 1.5–17.5 %); median interval between detection by DS and detection by conventional sequencing was 3 months (range, 1–9 months). In 5 cases, the mutations were detectable at baseline. In the remaining cases, response level at the time mutations were first detected by DS could be defined as ‘Warning’ (according to the 2013 ELN definitions of response to 2nd-line therapy) in 13 cases, as ‘Optimal response’ in one case, as ‘Failure’ in 4 cases. No dasatinib- or nilotinib-resistant mutations were detected by DS in 15 randomly selected patients with ‘warning’ at various timepoints, that later turned into optimal responders with no treatment changes.

**Conclusions:**

DS enables a larger window of detection of emerging BCR-ABL1 KD mutations predicting for an impending relapse. A ‘Warning’ response may represent a rational trigger, besides ‘Failure’, for DS-based mutation screening in CML patients undergoing second-line TKI therapy.

## Background

Several tyrosine kinase inhibitors (TKIs) can effectively target the BCR-ABL1 oncoprotein resulting from the t(9;22) chromosomal translocation in chronic myeloid leukemia (CML) patients. However, resistance continues to be a significant challenge in the management of CML. The acquisition of point mutations in the BCR-ABL1 kinase domain (KD) may undermine the efficacy of imatinib, and even second-generation TKIs (dasatinib, nilotinib, bosutinib) maintain a small but definite subset of resistant mutations [1]. Although dasatinib, nilotinib and bosutinib have demonstrated good efficacy in patients resistant to first-line TKI treatment with imatinib, approximately half of the patients experience a second relapse [2–4]. Increased expression and functional reactivation of BCR-ABL1 associated with resistance [5–7] foster genomic instability and perturbed differentiation, thus increasing the propensity to progress from chronic phase (CP) to blast crisis (BC) [8–10]. Even in the TKI era, treatment of BC remains a challenge and patients who progress have a dismal outcome: hence, preventing resistance as a mean to prevent disease progression from CP to BC is a crucial treatment endpoint [11, 12]. The percentage of patients positive for BCR-ABL1 KD mutations is approximately 30 % in case of resistance to first-line TKI treatment and rises up to 50–60 % in case of resistance to second-line TKI treatment [13]. In patients already harboring mutations selected by imatinib treatment, acquisition of new mutations conferring resistance to second-line therapy may give rise to very aggressive multi-mutated clones (‘compound mutants’) that are very difficult to counteract [14, 15]. These evidences indicate that CML patients receiving second-line TKI treatment are a critical subset: they have a higher risk of disease relapse and progression and not infrequently BCR-ABL1 KD mutations are implicated in therapeutic failure. In this setting, earlier detection of emerging BCR-ABL1 mutations would therefore be valuable to enable a greater leeway in tackling resistance, thus enhancing the efficacy of salvage therapy.

We have recently set up an assay for next generation amplicon-based deep sequencing (DS) of the BCR-ABL1 KD and have validated its accuracy, precision, and linearity for detection of any sequence variation down to 1 % [16, 17]. DS might be a reliable and sensitive candidate alternative to conventional sequencing, currently used for routine BCR-ABL1 KD mutation screening [18, 19]. We thus aimed to assess whether, and in how many patients receiving second-line TKI therapy, DS may identify clinically actionable TKI-resistant mutations earlier than conventional sequencing.

## Methods

### Patients and experimental design

Among the imatinib-resistant CML patients who switched to second-line TKI therapy and were referred to our laboratory for routine BCR-ABL1 transcript level monitoring and KD mutation screening, 51 later acquired dasatinib-(*n* = 26) or nilotinib-resistant mutations (*n* = 25) detected by conventional sequencing at the time of Failure, after a median of 9 months (range, 3–27 months) of therapy (Table 1). DS reanalysis was performed from the time of failure and mutation detection by conventional sequencing backwards. A total of 125 peripheral blood samples were studied. For comparison, 15 randomly selected patients with ‘Warning’ response at various timepoints, that later turned into stable ‘Optimal’ responses without treatment changes, were also analyzed by DS. No patient with suspected or confirmed lack of adherence, as well as no patient who had experienced dose adjustments or temporary discontinuations for toxicity was included in either group. The study was approved by the Institutional Review Board of the S. Orsola-Malpighi Hospital (study code 253/2013/O) and was conducted in accordance with the Declaration of Helsinki. Written informed consent for participation in this study was obtained from all the patients. The results of this study have been presented in abstract form at the 56^th^ annual meeting of the American Society of Hematology (ASH) in San Francisco (CA) in December 2014.

**Table 1:** Patients’ characteristics

Pts, total	51
CP CML	33
AP/BC CML	18
- with baseline IM-resistant mutations	29
- who acquired DAS-resistant mutations	26
T315I	13
F317L/V	10
V299L	3
- who acquired NIL-resistant mutations^a^	25
E255K/V	9
F359V/I/C	7
Y253H	6
T315I	4
Median time on 2nd-line therapy, months (range)	9 (3–27)

*Abbreviations*: *CP* chronic phase (at the time of second-line TKI therapy start), *AP/BC*, accelerated phase or blast crisis (at the time of second-line TKI therapy start), *IM* imatinib, *DAS* dasatinib, *NIL* nilotinib, the ^a^ denotes that one patient had two mutations

### BCR-ABL1 transcript level monitoring by real time quantitative polymerase chain reaction (RQ-PCR)

BCR-ABL1/ABL1% transcript levels were assessed by real time quantitative reverse transcription polymerase chain reaction (RQ-PCR) as previously described [20] and were expressed on the International Scale (IS) [21].

### Conventional sanger sequencing

Conventional sequencing of the BCR-ABL1 KD, amplified by nested RT-PCR, was performed according to the Sanger method on an ABI PRISM 3730 (Applied Biosystems, Foster City, CA) as previously reported [22, 23].

### Deep sequencing

The detailed DS protocol has been previously published [16]. Briefly, four amplicons spanning the BCR-ABL1 KD, tagged with a 10-base ‘barcode’ sequence (multiplex identifier), were generated by nested reverse transcription polymerase chain reaction and pooled in equimolecular ratios. DS was performed on a GS Junior instrument (Roche) according to the manufacturer’s instructions. Sensitivity, accuracy and reproducibility of our DS-based BCR-ABL1 mutation screening assay have already been demonstrated, as described in [16]. Minimum sequencing depth was 5,000x, ensuring detection of variants down to 1 %. Amplicon Variant Analyzer ver2.7 (Roche) was used to align reads to the reference ABL1 sequence (GenBank accession no.X16416.1) and to calculate variant frequencies. The presence of all relevant mutations was also manually verified by inspection of individual flowgrams at the corresponding positions, with particular attention to homopolymeric regions where sequencing errors tend to be more frequent.

### Response definitions

BCR-ABL1/ABL1% transcript levels were used to define whether the patient had an ‘Optimal response’, ‘Warning reponse’ or ‘Failure response’ at the time of first mutation detection by DS, according to the 2013 ELN recommendations [24].

## Results

**Fig. 1:**
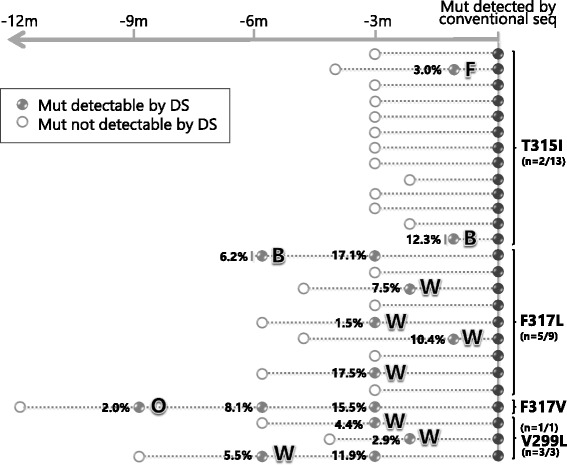
Backtracking dasatinib-resistant mutations by DS. Each line represents a patient and each circle corresponds to a sample. Full and empty circles indicate samples with mutations detectable or undetectable by DS, respectively. Light grey filling denotes samples in which the mutation was detectable by DS only. Dark grey filling denotes samples in which the mutation was detectable also by conventional sequencing. For each type of mutation, numbers in parentheses summarize the number of patients in which the mutation could be backtracked by DS/the total number of patients who acquired that type of mutation. Percentages indicate mutation relative abundance. ‘F’ means ‘Failure’, ‘W’ means ‘Warning’, ‘O’ means ‘Optimal’ response; ‘B’ means ‘Baseline’

Among the 26 patients who relapsed on dasatinib, 13 had acquired a T315I mutation, 10 had acquired F317L or V mutations, and 3 had acquired a V299L mutation (Fig. 1). DS allowed to backtrack mutations in 11 cases (T315I, *n* = 2; F317L/V, *n* = 6; V299L, *n* = 3). In 2 patients, the mutations were detected at baseline. In the remaining cases, correlation with response at the time mutations were first detected by DS revealed a ‘Warning’ in 7 cases; a ‘Failure’ in 1 case; an ‘Optimal response’ in 1 case (Fig. 1).

**Fig. 2:**
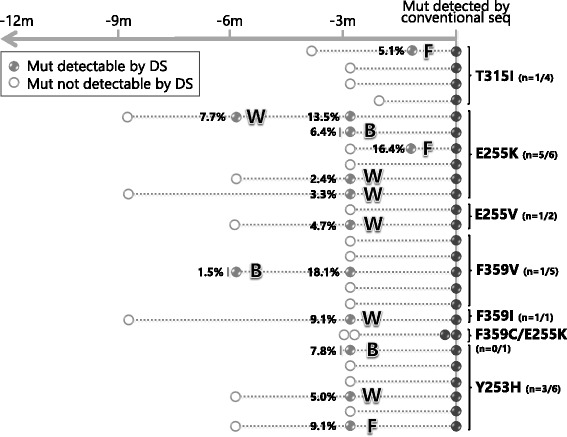
Backtracking nilotinib-resistant mutations by DS. See legend to Fig. 1 for explanations and abbreviations

Among the 25 patients who relapsed on nilotinib, 4 had acquired a T315I mutation, 8 had acquired an E255K or V mutation, 6 had acquired an F359V or I mutation, 1 had acquired an F359C and an E255K simultaneously, and 6 had acquired a Y253H mutation (Fig. 2). DS allowed to backtrack mutations in 12 cases (T315I, *n* = 1; E255K/V, *n* = 6; F359V/I, *n* = 2; Y253H, *n* = 3). In 3 cases, the mutations were detected at baseline. In the remaining patients, response levels at the time mutations were first detected by DS were: ‘Warning’ in 6 cases; ‘Failure’ in 3 cases (Fig. 2).

Thus, overall, DS could detect emerging BCR-ABL1 mutants earlier than conventional sequencing (median, 3 months; range, 1–9 months) in 23/51 (45 %) cases. Median mutation burden at the time of first detection by DS was 5.5 % (range, 1.5 %–17.5 %).

We next checked if low level mutations can be identified in cases with ‘Warning’ responses who ultimately become optimal responders. To address this issue, DS was also performed, for comparison, in 15 randomly selected patients with ‘Warning’ response at various timepoints, that later turned into stable ‘Optimal’ responses without treatment changes. Reassuringly, no low-level TKI-resistant mutations that would have triggered an unnecessary treatment change were detected by DS.

Finally, we checked how many of the 28 patients in whom DS failed to detect the emerging mutation(s) in the earlier sample had a ‘Failure’ or ‘Warning’ response level at that time – to estimate in how many cases DS would be performed without bringing any advantage over conventional sequencing. In the dasatinib group, 15 patients had no mutations detectable by DS in the sample immediately before (most frequently, 3 months before) conventional sequencing testing. At the corresponding timepoint, 1 patient had a response level already classifiable as Failure, 3 patients had a ‘Warning’ response and 11 patients had an ‘Optimal response’. In the nilotinib group, patients in whom DS failed to detect the mutation earlier were 13. Two of them had a ‘Warning’ and 11 had an ‘Optimal response’. So, in our series, only six cases would have had longitudinal testing by DS with no earlier detection of the emerging mutation.

## Conclusions

Imatinib-resistant CML patients receiving second-line TKI therapy may develop new mutations leading to a second relapse. Despite availability of several TKI options, salvage rates for these patients remain pretty unsatisfactory [25, 26]. Our results indicate that DS enables a larger window of detection of emerging BCR-ABL1 KD mutations predicting for an impending relapse. Earlier detection of a mutation known to confer resistance to the TKI the patient is receiving may offer greater chances of efficacy for subsequent salvage therapy and limit the biological consequences of full BCR-ABL1 kinase reactivation.

In order to identify patients with emerging mutations, when should DS analysis be performed? Regular surveillance of BCR-ABL1 KD sequences by DS in all patients on second-line therapy, in parallel with RQ-PCR monitoring, would not probably be cost-effective. The 2013 ELN treatment recommendations [24] have established critical checkpoints and definite BCR-ABL1 transcript level thresholds to define three response categories – ‘Failure’ (the patient should receive a different treatment to limit the risk of progression and death), ‘Warning’ (more frequent monitoring is needed to permit timely change in therapy in case of treatment failure) and ‘Optimal’ response (there is no indication for a change in treatment). In CML patients on second-line TKI therapy, BCR-ABL1 KD mutation analysis by conventional sequencing is currently recommended at baseline and the time of ‘Failure’, when it may provide important information to be included in the therapeutic decision algorithms [18]. The results of this study provide further confirmation that DS of the BCR-ABL1 KD at baseline and at the time of ‘Failure’ would detect mutations in a greater proportion of patients as compared to conventional sequencing and would better inform therapeutic choices [27]. More importantly, our findings suggest that during second-line TKI therapy, DS may identify emerging mutations earlier than conventional sequencing. A ‘Warning’ response may represent, besides ‘Failure’, a reasonable trigger for the application of DS-based mutation screening. In thirteen cases, low level mutations resistant to the ongoing TKI were retrospectively detected by DS when response was still at the level of ‘Warning’ and not yet at the level of ‘Failure’. In many patients ‘Warning’ is a transient condition, that may later turn into ‘Failure’ or, in some cases, into an ‘Optimal’ response. To rule out the possibility that, in some cases, low level mutations resistant to the ongoing TKI may be a transient finding and may not always correlate with subsequent treatment failure, we randomly selected 15 patients with ‘Warning’ response that later became stable optimal responders. DS analysis of the samples collected at the time of ‘Warning’ in these patients did not show evidence of low level mutations. This demonstrates that detection of low burden mutations known to confer resistance to the TKI the patient is receiving can reasonably be considered a reliable indication for treatment change in all cases with a ‘Warning’ response.

This study thus provides further evidence of how clinical actionability may be enhanced by routine DS-based BCR-ABL1 KD mutation screening and comes at a turning point witnessing a gradual transition from conventional to next-generation sequencing for the diagnostic assessment of disease (and cancer)-related genes [28]. It also contributes to build the background for implementing technical and clinical recommendations for CML monitoring and management.

## Abbreviations

BC, blast crisis; CML, chronic myeloid leukemia; CP, chronic phase; DS, deep sequencing; IS, International Scale; KD, kinase domain; RQ-PCR, real time quantitative reverse transcription polymerase chain reaction; TKIs, tyrosine kinase inhibitors
